# *CERTOMICS*: trusted single-cell multiomics pipeline for high-resolution profiling of adoptive cellular immunotherapies

**DOI:** 10.1093/bioinformatics/btag096

**Published:** 2026-02-25

**Authors:** Christina Katharina Kuhn, David Schmidt, Michael Rade, Josephine Selke, U Sandy Tretbar, Maximilian Merz, Jan Grau, Kristin Reiche

**Affiliations:** Department of Medical Bioinformatics, Fraunhofer Institute for Cell Therapy and Immunology IZI, Leipzig 04103, Germany; Fraunhofer Cluster of Excellence Immune-Mediated Diseases, Leipzig 04103, Germany; Department of Medical Bioinformatics, Fraunhofer Institute for Cell Therapy and Immunology IZI, Leipzig 04103, Germany; Fraunhofer Cluster of Excellence Immune-Mediated Diseases, Leipzig 04103, Germany; Department of Medical Bioinformatics, Fraunhofer Institute for Cell Therapy and Immunology IZI, Leipzig 04103, Germany; Fraunhofer Cluster of Excellence Immune-Mediated Diseases, Leipzig 04103, Germany; Department of Medical Bioinformatics, Fraunhofer Institute for Cell Therapy and Immunology IZI, Leipzig 04103, Germany; Department of Medical Bioinformatics, Fraunhofer Institute for Cell Therapy and Immunology IZI, Leipzig 04103, Germany; Fraunhofer Cluster of Excellence Immune-Mediated Diseases, Leipzig 04103, Germany; Department of Medical Bioinformatics, Fraunhofer Institute for Cell Therapy and Immunology IZI, Leipzig 04103, Germany; Department of Hematology, Cell Therapy and Hemostaseology, University Hospital of Leipzig, Leipzig 04103, Germany; Myeloma Service, Memorial Sloan Kettering Cancer Center, New York, NY 10065, United States; Cancer Center Central Germany (CCCG) Leipzig-Jena, University Hospital of Leipzig, Leipzig 04103, Germany; Institute of Computer Science, Martin Luther University Halle-Wittenberg, Halle (Saale) 06099, Germany; Department of Medical Bioinformatics, Fraunhofer Institute for Cell Therapy and Immunology IZI, Leipzig 04103, Germany; Fraunhofer Cluster of Excellence Immune-Mediated Diseases, Leipzig 04103, Germany; Department of Hematology, Cell Therapy and Hemostaseology, University Hospital of Leipzig, Leipzig 04103, Germany; Cancer Center Central Germany (CCCG) Leipzig-Jena, University Hospital of Leipzig, Leipzig 04103, Germany; Center for Scalable Data Analytics and Artificial Intelligence (ScaDS.AI), Leipzig 04105, Germany; Interdisciplinary Transformation University IT:U, Linz 4020, Austria

## Abstract

**Summary:**

Adoptive cellular immunontherapies, such as chimeric antigen receptor (CAR) T cell therapy, have transformed cancer treatment, yet challenges such as resistance, relapse, and high costs limit their efficacy and accessibility. A comprehensive understanding of cellular heterogeneity and molecular profiles is essential to improve these therapies. Advanced single-cell multiomics technologies have the power to analyze the complex interactions between CAR-engineered cells, immune cells, and tumor cells. However, standardized single-cell multiomics computational pipelines specifically tailored to CAR-engineered cell products are lacking. Due to the synthetic nature of CAR transgenes, additional steps for reliable identification and characterization of CAR-positive cells are required but not included in existing data-processing workflows. To address this, we present *CERTOMICS*, a Nextflow-based, CAR-aware pipeline offering enhanced **CERT**ainty in immunophenotyping and data interpretation, tailored for single-cell multi**OMICS**profiling of adoptive cellular immunotherapies. The pipeline standardizes processing 10x Genomics single-cell multiomics data and integrates CAR-specific identification and quality control. Additionally, a curated repository of CAR construct sequences and annotation data is provided, serving as an extensible resource to support the analysis and development of CAR T cell therapies.

**Availability and implementation:**

Detailed documentation of this pipeline, along with a resource on latest FDA-approved CAR therapies is available on our website: https://fraunhofer-izi.github.io/Living-Drugs-Wiki/. The data underlying this article are available on GitHub at https://github.com/fraunhofer-izi/CERTOMICS. The code is also published on Zenodo at https://doi.org/10.5281/zenodo.18709693.

## 1 Introduction

Adoptive cellular immunotherapies, including chimeric antigen receptor (CAR) T cell therapies, have emerged as a successful treatment option for hematological cancers and beyond (Cappell and Kochenderfer 2023, Uslu and June 2025). These therapies involve engineering immune cells with synthetic CAR receptors, enabling them to recognize and eliminate cancer cells. Since 2017, seven CAR T cell therapies have been approved by the FDA for the treatment of blood cancers, including multiple myeloma, B cell leukemia, and lymphoma (Sengsayadeth *et al.* 2021, US Food and Drug Administration 2024). Despite their promise, CAR T cell therapies still face challenges, including high costs, disease relapse, antigen escape, severe side effects and suppression by the tumor microenvironment (TME) in solid tumors (Chen *et al.* 2023). To address these limitations, there is growing interest in engineering other immune cell types—such as natural killer (NK) cells and macrophages—with CAR constructs, expanding the therapeutic potential of adoptive cell therapy (Klichinsky *et al.* 2020, Zhang *et al.* 2024).

As a “living drug,” CAR-engineered immunotherapies rely on the dynamic and evolving nature of engineered immune cells within the patient. Consequently, a comprehensive characterization of both (CAR-engineered) immune cell heterogeneity, the target cells, and the disease-specific cellular and molecular environment is crucial for understanding their functional diversity, persistence, and therapeutic efficacy. Advanced single-cell multiomics technologies provide a powerful tool to analyze the complex interactions between engineered cells, endogenous immune populations, and tumor cells (Deng *et al.* 2020, Sheih *et al.* 2020, Huang et al. 2023). Beyond single-cell gene expression profiling (GEX), established assays (e.g. from 10× Genomics or other vendors) enable researchers to simultaneously sequence T and B cell receptors via V(D)J sequencing (variable [V], diversity [D], and joining [J] segments) and detect cell surface proteins using antibody-derived tags (ADT). The integration of these techniques is particularly advantageous for the characterization of the transcriptional profiles of immune cell populations, the assessment of clonal expansion of (CAR) T cells, and the identification of outcome-associated markers (Rade *et al.* 2024, Braun *et al.* 2025). High-quality single-cell multiomics data are essential for constructing patient-specific virtual twins in engineered adoptive cellular immunotherapies (Reiche *et al.* 2025, Weirauch *et al.* 2025). However, standardized computational workflows for multiomics single-cell profiling of CAR-engineered cell products are lacking. Given the synthetic nature of CAR transgenes, additional reference genome building and specialized quality control (QC) steps are essential to reliably detect and characterize CAR-positive cells. While *scFlow* (Khozoie *et al.* 2021), *scDrake* (Kubovčiak et al. 2023), and *Panpipes* (Curion *et al.* 2024) do not include CAR-specific functionality, *scFlow* and *scDrake* additionally lack support for the integrated analysis of multimodal datasets, such as ADT-seq or V(D)J-seq. Furthermore, *Panpipes* and *scDrake* are not implemented in Nextflow, which limits its scalability across computational environments, reproducibility, and compliance with modern workflow standards. Overall, these limitations hinder comprehensive and standardized single-cell multiomics analyses of CAR-engineered immune cells. To address these challenges, we present *CERTOMICS*, a Nextflow-based pipeline designed for standardized, scalable, and user-friendly single-cell multiomics profiling of CAR-engineered immune cells. In contrast to existing single-cell workflows, *CERTOMICS* combines multi-modal processing and QC [GEX, V(D)J, ADT] with CAR-aware reference construction and CAR-specific QC. Its modular Nextflow architecture enables extensibility and interoperability, allowing users to incorporate custom analytical steps without modifying the core pipeline. Together, these features ensure robust detection and characterization of CAR-positive cell populations and support seamless end-to-end data integration into an annotated Seurat object. Beyond the pipeline, a comprehensive, curated repository of the sequences of CAR T cell products is provided on GitHub, containing nucleotide sequences and annotations of synthetic CAR constructs, facilitating seamless detection and characterization of CAR-positive cells within single-cell multiomics datasets. By offering a streamlined computational workflow and a well-annotated CAR construct sequence database, *CERTOMICS* enhances in-depth characterization of adoptive cellular immunotherapies, and supports the continued advancement of CAR-engineered cell therapies.

## 2 CAR-specific features

**Figure 1:**
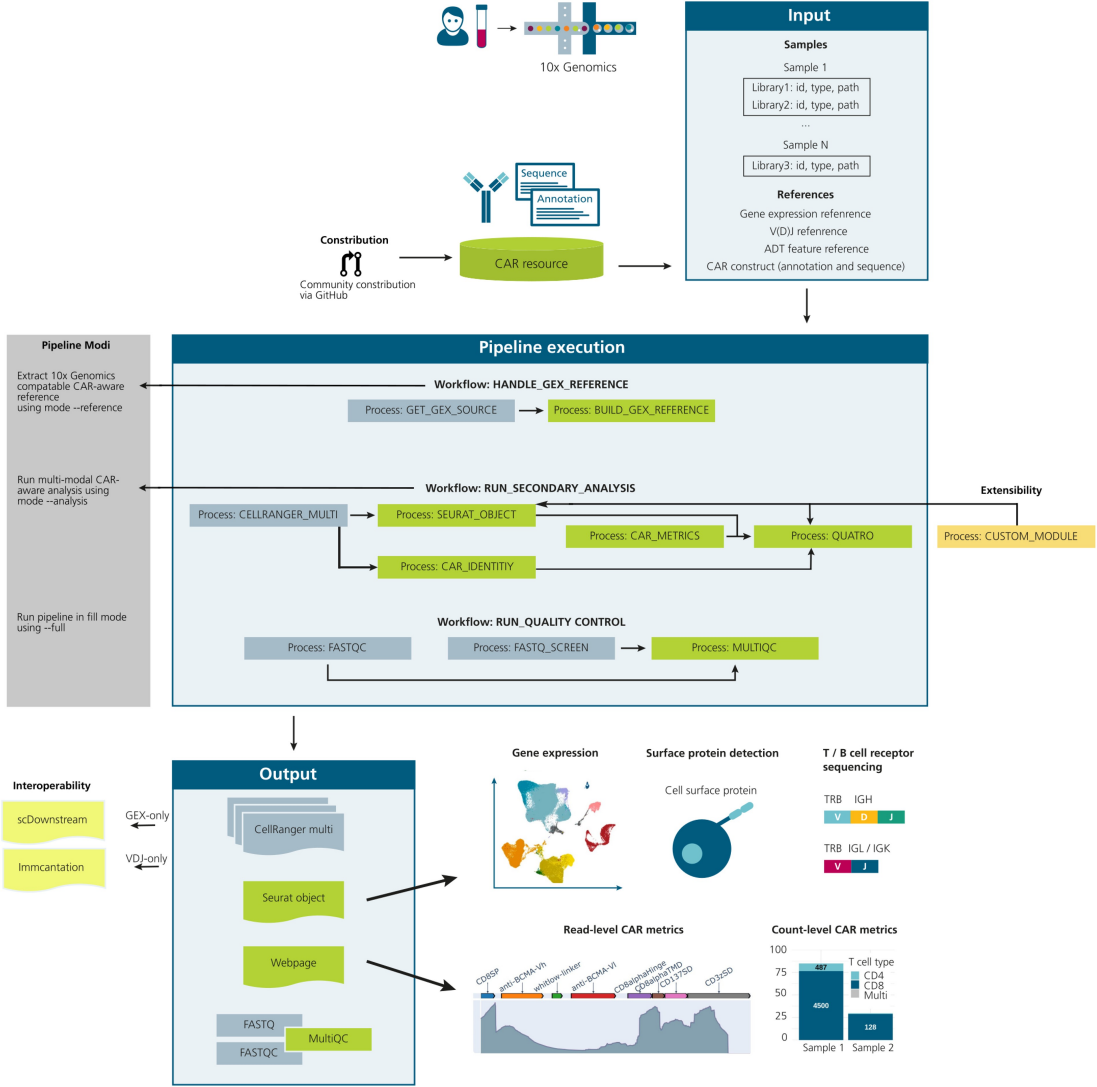
Overview of the *CERTOMICS* pipeline for single-cell multiomics analysis including CAR T cell products. A set of samples (Sample1 to SampleN), each with associated sequencing libraries—gene expression (GEX), T cell receptor profiling [V(D)J], and antibody-derived tags (ADT)—as well as CAR construct data (FASTA and GTF) are processed. CERTOMICS first builds a CAR-aware reference (HANDLE_GEX_REFERENCE), processes multi-modal sequencing data (RUN_SECONDARY_ANALYSIS) into a merged, annotated Seurat object, and generates an interactive results webpage including CAR-metrics. Users can optionally enable RUN_QUALITY_CONTROL to perform multi-modal QC via FastQC, FASTQ_Screen, and MultiQC. The pipeline supports three execution modes (--reference, --analysis, and --full), allowing modular use of individual components. Contribution to the CAR resource (GitHub symbol), extensibility of the pipeline, and interoperability with external tools are highlighted.

Unlike standard single-cell multiomics sequencing workflows, *CERTOMICS* offers several features specifically tailored to CAR-engineered cell products (highlighted in Fig. 1), while also enabling integration of multiomics data. To enable a deep characterization of CAR-engineered cell products, *CERTOMICS* supports the integration of various combinations of gene expression and V(D)J libraries, with or without feature barcode libraries, across multiple samples. Specifically, it supports the analysis of common 10x Genomics single-cell (immune profiling) libraries using CellRanger Multi. A custom multi-modal MultiQC module facilitates cross-sample assessment of sequencing quality and modality integration (Figs 1 and 2, available as supplementary data at *Bioinformatics* online) (Ewels *et al.* 2016). A key feature of the pipeline is its ability to detect CAR-positive cells by incorporating a dedicated reference processing step. For this step, the user simply provides a CAR sequence file (.fasta) and CAR annotation file (.gtf). The pipeline offers curated CAR sequences for several approved CAR T cell therapies (see section Resource on CAR T cell products). The reference-building process supports both publicly available 10x Genomics human reference builds from 2024 to 2020, which can be specified using the—gene_expression_reference_version parameter in the -params-file. However, the pipeline also allows the use of an own 10x compatible prebuilt reference—gene_expression_reference parameter.

### 2.1 Merged seurat object

Beyond generating standard per-sample output directories from CellRanger Multi, the pipeline integrates all processed samples into a merged Seurat object (seurat_merged.Rds) (Hao et al. 2023). This object consolidates data from multiple library types [GEX, V(D)J, ADT] and extends it with comprehensive metadata, enabling streamlined downstream analysis. The metadata includes: quality metrics (mitochondrial and ribosomal gene abundance, cell cycle scores, and doublet removal information), cell type annotations based on scGate, where the gating model [peripheral blood mononuclear cells (PBMC) or high-resolution TME] can be specified by the user (Andreatta *et al.* 2022), and integrated clonotype information derived from V(D)J sequencing. This merged dataset offers a comprehensive and structured representation of the processed samples, enabling in-depth downstream analysis and detailed characterization of CAR T cell products across multiple samples. Detailed processing of CellRanger output and Seurat object generation is described in Methods, available as supplementary data at *Bioinformatics* online.

### 2.2 Interactive quality control reports

Additionally, the pipeline generates an interactive webpage that consolidates cross-sample and CAR-specific quality metrics, exemplified using a publicly available longitudinal BCMA-directed CAR T cell dataset(Figs 3 and 4, available as supplementary data at *Bioinformatics* online) (Rade *et al.* 2025). CAR-specific metrics are derived from two levels of data: “Read-level” metrics, based on mapped sequencing reads and “Count-level” metrics, based on CellRanger raw counts (Fig. 3, available as supplementary data at *Bioinformatics* online).

- **Read-level metrics** include coverage plots across the CAR construct, enabling assessment of read distribution and sequencing protocol performance ($5^{\prime }$ or $3^{\prime }$), as well as absolute read counts per sample to evaluate transgene expression. Additionally, an optional validation step can be performed to confirm correct CAR construct identity by comparison to alternative CAR constructs, serving as internal controls to assess detection specificity
- **Count-level metrics** provide biological insights by quantifying CAR-positive cell frequencies in different immune cell populations. This allows the assessment of patient-specific variability or comparison across different condition, e.g. before versus after CAR expansion, as well as a negative control from CAR-negative cell populations.

In addition to CAR-specific quality control, the webpage presents general GEX-specific statistics, including cell proportions, as well as V(D)J sequencing statistics, such as clonotype composition for both T and B cells (Fig. 4, available as supplementary data at *Bioinformatics* online). These additional metrics provide insights into TCR/BCR diversity, expansion patterns, and immune repertoire changes, which are critical for understanding persistence, functionality, and immune cell interactions. Summary report generation is described in Methods, available as supplementary data at *Bioinformatics* online (“Summary webpage construction”).

## 3 Resource on CAR T cell products

There are significant differences between CAR constructs used in various CAR T cell products (see Fig. 5, available as supplementary data at *Bioinformatics* online). Precise knowledge of CAR nucleotide sequences is essential for sequence analyses, including accurate identification of CAR expression, mutations, binding affinities, and evaluation of immunogenicity—all critical for optimizing CAR design and functionality. Furthermore, the choice of gene transfer vector used for transduction can affect the efficiency, safety, and stability of gene transfer and affects gene expression (Morgan and Boyerinas 2016, Rad *et al.* 2020, Ho *et al.* 2021). Therefore, we provide a resource designed to support the analysis and development of CAR T cell therapies by offering comprehensive nucleotide sequence and annotation data on currently available CAR constructs (Resources/CAR_constructs) and vector systems (Resources/Vector_systems). Sequence information of CAR T cell products has been collected from literature and patents, assembled as FASTA files, and complemented with domain annotation, assembled as GTF files. The retrieval and annotation process, as well as the original sources (Table 1, available as supplementary data at *Bioinformatics* online), are described in Methods, available as supplementary data at *Bioinformatics* online “Retrieval of nucleotide sequences.” The complete resource can be viewed at our webpage: https://fraunhofer-izi.github.io/Living-Drugs-Wiki/Home/Resources/. In combination with *CERTOMICS*, this resource enables users to seamlessly detect CAR-positive T cells within their single-cell multiomics data. While over 2000 CAR T or engineered cellular immunotherapy clinical trials are registered (ClinicalTrials.gov. (2025, August 8). *Search results for: (engineered cellular immunotherapy) OR (CAR T)*. U.S. National Library of Medicine. Retrieved from https://clinicaltrials.gov/), the corresponding CAR nucleotide sequences are often not yet available through public patent applications. Users of *CERTOMICS* interested in incorporating a CAR construct not yet included in our resource can use the parameters --gene_expression_source_fa and --gene_expression_source_gtf. The resource is openly developed on GitHub, and users can contribute new CAR constructs through a structured workflow ensuring consistent annotation quality.

## 4 Implementation

The pipeline is implemented in Nextflow (Tommaso *et al.* 2017) (version ≥ 4.0.12) and supports execution via Singularity containers for reproducability (Kurtzer *et al.* 2017). It is structured into three main processes: (i) handling raw data and references for different libraries, (ii) performing core secondary analysis, and (iii) running optional quality control.

1. HANDLE_REFERENCES—Generates custom reference files for CellRanger based on the sequencing libraries [GEX, V(D)J, ADT] and the CAR construct (FASTA, GTF) given.
2. RUN_SECONDARY_ANALYSIS—Executes CellRanger multi and generates a merged, annotated Seurat object, and computes CAR-specific quality control metrics, with results summarized in a dedicated webpage.
3. RUN_QUALITY_CONTROL—Conducts quality control assessments using FASTQC (v0.12.1) FASTQ Screen (v0.15.3), and a custom multi-modal MultiQC (v1.24.1) module to evaluate quality and composition of a multi-modal single-cell sequencing experiment.

Execution environment are managed through predefined profiles (-profile), enabling adaptation to specific infrastructures. Specifically, it supports the workload manager Slurm for efficient process distribution in HPC environments while also providing a profile for local execution (Jette *et al*. 2023). User input is provided via a YAML or JSON file that defines the required parameters. This file, specified using the -params-file flag, contains essential information such as input data (references, samples, and CAR information) and optional settings, such as the output directory. The required references and sample details depend on the sequencing libraries generated [GEX, V(D)J, or ADT], and users should include only those relevant to their experiment.

The main command for running the pipeline is:

nextflow run main.nf

-profile < profile(-s) >

-params-file < your params-file >

Design principles for modularity and extensibility

As *CERTOMICS* is implemented with Nextflow it comes with modularity as a core design principle. Reference handling and multi-modal secondary analysis including CAR-specific QC are implemented as independent Nextflow processes and can be runned via different execution modes (see Fig. 1). This structure enables users to:

1. execute only selected modules (e.g. using *CERTOMICS* solely for CAR-aware reference generation),
2. integrate modality-specific outputs (e.g. GEX-only) into other pipelines such as scDownstream, and
3. extend the pipeline with custom user-defined modules.

## 5 Conclusion

*CERTOMICS* offers a standardized framework for characterizing biospecimens containing CAR-engineered cell products. By simultaneously profiling gene expression, immune repertoire, and cell surface proteins, it is the first standardized pipeline to provide a high-resolution view of cellular heterogeneity in engineered immune cells such as CAR T cells. The pipeline enables precise detection and profiling of CAR-positive cells and their interactions with tumor and host immune cells by generating a CAR-aware reference genome and incorporating CAR-specific quality control metrics. To ensure reliability, the CAR reference resource will be continuously updated to reflect the diversity of constructs emerging from clinical research.

The integration of single-cell multi-modal data into a unified Seurat object, combined with a custom MultiQC module, supports efficient and reproducible in-depth analyses. Interactive summary reports presenting CAR-, GEX-, and V(D)J-specific metrics further enhance interpretability and facilitate robust cross-sample comparisons. The standardized implementation in Nextflow ensures reproducibility, scalability, and ease of deployment across diverse computing environments. Beyond standardization, *CERTOMICS* uniquely integrates three elements not combined in any existing workflow: (i) multi-modal single-cell processing, (ii) automated CAR-aware reference construction using a curated and continuously updated CAR sequence repository, and (iii) a modular, extensible Nextflow architecture supporting user-defined analyses and interoperability with other pipelines. This combination establishes *CERTOMICS* as more than a standard pipeline, providing a flexible framework that can evolve with emerging CAR designs, sequencing protocols, and research questions.

As engineered adoptive cellular immunotherapies expand beyond hematologic malignancies into autoimmune diseases and solid tumors (Baker *et al.* 2023, Chung *et al.* 2024, Keitel *et al.* 2025), the need for standardized, high-quality single-cell characterization becomes increasingly critical (Kirtane *et al.* 2021, Weirauch *et al.* 2025). *CERTOMICS* addresses this need by providing a reproducible, quality-controlled, and scalable framework for multiomics profiling of CAR-engineered products. While currently optimized for the most widely used 10x Genomics protocols, it is designed to be adaptable in future versions to support emerging technologies such as the GEM Flex assay, ATAC-seq, and long-read sequencing or even going beyond genetically engineered CAR T cells also covering CAR NK/macrophages or emerging new technologies of engineering immune cells to fight diseases.

## Supplementary Material

btag096_Supplementary_Data
