# Supplementary material for: CERTOMICS: trusted single-cell multiomics pipeline for high-resolution profiling of adoptive cellular immunotherapies
Source: Bioinformatics. 2026 Feb 25;42(3):btag096. doi: 10.1093/bioinformatics/btag096 (PMC13008325; doi:10.1093/bioinformatics/btag096)
Supplement: btag096_Supplementary_Data [file btag096_supplementary_data.zip › Supplementary.pdf]

, , pp. 1–9

doi:

Suplementary Data for "*CERTOMICS*: Trusted Single-Cell Multiomics Pipeline for High-Resolution Profiling of Adoptive Cellular Immunotherapies"

Christina Katharina Kuhn[1,*] David Schmidt,[1] Michael Rade,[1] Josephine Selke,[1] U. Sandy Tretbar,[1] Maximilian Merz,[1,3,4,5] Jan Grau[2] and Kristin Reiche[1,3,5,6,7,8]

[1]Department of Medical Bioinformatics, Fraunhofer Institute for Cell Therapy and Immunology IZI, 04103, Leipzig, Germany, [2]Institute of Computer Science, Martin Luther University Halle-Wittenberg, 06099, Halle (Saale), Germany, [3]Department of Hematology, Cell Therapy and Hemostaseology, University Hospital of Leipzig, 04103, Leipzig, Germany, [4]Myeloma Service, Memorial Sloan Kettering Cancer Center, 10065 New York, USA, [5]Cancer Center Central Germany (CCCG) Leipzig-Jena, University Hospital of Leipzig, 04103, Leipzig, Germany, [6]Center for Scalable Data Analytics and Artificial Intelligence (ScaDS.AI), Dresden, Leipzig, Germany, [7]Interdisciplinary Transformation University IT:U, 4020, Linz, Austria and [8]Fraunhofer Cluster of Excellence Immune-Mediated Diseases, 04103, Leipzig, Germany

*Corresponding author. christina.kuhn@izi.fraunhofer.de

Supplementary Methods

Processing of Cellranger output

A custom R script (v4.4.2) was developed using Seurat (v5.1.0) (Hao et al. 2023) to process 10x Genomics gene expression (GEX) data and, when available, antibody-derived tag (ADT) data from the CellRanger (v8.0.1) output (Zheng et al. 2017). To account for ambient RNA contamination, the R package SoupX v1.6.2 (Young and Behjati 2020) was applied. Corrected raw counts generated by SoupX were subsequently used. Doublet removal scores were computed and annotated in the metadata using the `scDblFinder` R package (v1.20.0) (Germain et al. 2022). Raw gene expression data was normalized using the LogNormalize method of the Seurat `NormalizeData` function. ADT data was normalized by the centered log-ratio transformation (CLR) with parameters normalization.method = "CLR" and default margin. Cell-specific quality control (QC) metrics were calculated and added as metadata, including mitochondrial gene abundance, ribosomal gene abundance, and cell complexity, which was defined as:

$$\log_{10}(\text{number of detected genes})/\log_{10}(\text{number of UMIs})$$

Cells that met any of the following criteria are flagged with a filtering parameter (`KEEP_CELL == FALSE`), although all cells remain in the Seurat object. This minimal filtering approach is primarily intended for generating the summary webpage, but it may not be optimal for all experimental contexts and should be adapted as needed: (1) fewer than 250 genes; (2) more than 8,000 genes; (3) fewer than 1,000 UMIs; (4) more than 10,000 UMIs; (5) mitochondrial transcript fraction greater than 15%; (6) cell complexity less than 0.8; or (7) cells identified as "doublets".

To identify proliferating cells (stored as `CellCycle_Phase`), we employed a two-step approach. First, initial cell cycle phase classifications were assigned using Seurat's `CellCycleScoring` function based on S- and G2M-phase gene sets from (Tirosh et al. 2016). Next, cluster-level cell cycle enrichment was determined using Gene Set Enrichment Analysis (GSEA) via the `run_gsea` function from the `clustifyr` package (V1.18.0) (Riemondy et al. 2020) identifying clusters significantly enriched in cycling genes (defined by ProjecTILs `cell.cycle.obj`) (Andreatta, Corria-Osorio, et al. 2021). To enhance sensitivity in detecting proliferative cell populations, an over-clustering approach was used (McGinnis et al. 2019). For this purpose, the cluster resolution was set to 1. For gene set enrichment, 1,000 permutations were performed, which is the default in standard GSEA workflows. One cell cluster was significantly (p-value < 0.05) enriched with cell cycle genes from the G2/M and S phases. Cells in these enriched clusters were assigned a cycling phase. Cells in non-enriched clusters retained their previous Seurat-based cell cycle annotations.

For cell type annotation, `scGate` (v1.6.2) was used, either with the PBMC gating model or the high-resolution TME model (TME_HiRes) and default parameters (Andreatta, Berenstein, et al. 2022).

Cell clonotypes information is added as metadata from V(D)J output using `scRepertoire` (v2.2.1) (Borcherding et al. 2020). Clonotypes were identified using the package's `combineTCR` and `combineBCR` functions across all samples. The combined TCR/BCR data is then integrated via using the package's `combineExpression` function. A clonotype is defined as the combination of genes comprising the V(D)JC gene plus CDR3 nucleotide (strict mode). Frequencies and proportions of clonotypes were calculated sample-wise.

Summary webpage construction

Based on the merged multi-modal Seurat object, summary webpage are generated and rendered with a quarto script (v1.6.40). Only cells passing a minimal pre-defined quality filter (`KEEP_CELL == TRUE`) (see Processing of Cellranger output) were used for plotting of CAR count-level, GEX-specific, and V(D)J-specific metrics. The number of cells per sample before and after filtering is reported in the summary page.

© The Author . Published by Oxford University Press. All rights reserved. For permissions, please e-mail: journals.permissions@oup.com

Read coverage and absolute read statistics are computed using Python (v3.10). Read coverage across the CAR construct is determined from uniquely and multimapping reads as identified by STAR alignments in the BAM output files. For the optional step to identify correct CAR construct compared to alternative CAR isoforms, construct specific 31-mers are generated and compared between mapped and unmapped reads. For mapped-read analysis, reads aligned to the CAR locus were extracted from the alignment BAM file and screened for the presence of CAR-specific unique k-mers to quantify construct-specific signal in confidently mapped reads. To capture CAR-derived fragments that fail alignment, all unmapped reads were extracted and indexed using KMC tools (Deorowicz et al. 2013) and intersections with CAR-specific k-mer sets were computed. Together, mapped and unmapped k-mer analyses provide a complementary validation for idenitity of CAR-transgene.

For CAR count-level metrics, absolute and relative number of CAR-positive cells were calculated based on scGate annotation. CAR-positive T cells were defined as all CD4, CD8 and gamma-delta T cells with CAR> 0 (raw counts) as defined as PURE according to scGate. GEX-specific metrics present cell type distributions as classified by `scGate_multi` parameter. For V(D)J-specific metrics absolute and relative numbers of unique clones are presented and highlighted by clone size grouping calculated with `combineExpression` from `scRepertoire`.

Retrieval of nucleotide sequences

Nucleotide sequence information was retrieved for several FDA-approved products (Axicel, Ciltacel (different sources), Idecel, Tisacel) via patent search and publication review. Original source of sequences and search query is described in Supplementary Table S1. A custom python script was developed to exact sequence information from PDF/PNG based on a custom template matching approach using structural similarity (SSIM) to identify sequence nucleotides. To ensure correct sequences, CAR constructs were manually re-checked subsequently. The same process was performed for complete CAR vector sequences, when available (Ciltacel, Idecel, Tisacel). All nucleotide sequences are provided in `FASTA` format.

Annotation of nucleotide sequences

In order to annotate structural components of CAR products, CAR nucleotide sequences where aligned to nucleotide sequences of known proteins, which the different CAR constructs are composed of (Figure S5). First, nucleotide sequences were obtained from NCBI (retrieval date: 22nd September 2025). Second, CAR nucleotide sequences where aligned to retrieve sequence position using `BLASTN` (from the NCBI BLAST+ suite v2.9.0+). Those annotations were complemented with known annotation from the original sources. For vector systems annotation was done using the Addgene online tool (`https://www.addgene.org/analyze-sequence/`). Features within `GTF` file were defined by using "Feature Type" according to Addgene "Feature Options" labels. Only the predicted open reading frame for the CAR construct was added. All annotation information are provided in `GTF` format.

References

Andreatta, Massimo, Ariel J. Berenstein, and Santiago J. Carmona (May 2022) "scGate: marker-based purification of cell types from heterogeneous single-cell RNA-seq datasets". In: *Bioinformatics (Oxford, England)* 38 (9) pp. 2642–2644.

Andreatta, Massimo, Jesus Corria-Osorio, et al. (May 2021) "Interpretation of T cell states from single-cell transcriptomics data using reference atlases". In: *Nature Communications 2021 12:1* 12 (1) pp. 1–19.

Borcherding, Nicholas, Nicholas L. Bormann, and Gloria Kraus (June 2020) "scRepertoire: An R-based toolkit for single-cell immune receptor analysis". In: *F1000Research 2020 9:47* 9, p. 47.

Deorowicz, Sebastian, Agnieszka Debudaj-Grabysz, and Szymon Grabowski (May 2013) "Disk-based k-mer counting on a PC" in: *BMC Bioinformatics 2013 14:1* 14 (1) pp. 160–.

Germain, Pierre Luc et al. (2022) "Doublet identification in single-cell sequencing data using scDblFinder". In: *F1000Research* 10.

Hao, Yuhan et al. (May 2023) "Dictionary learning for integrative, multimodal and scalable single-cell analysis". In: *Nature Biotechnology 2023 42:2* 42 (2) pp. 293–304.

McGinnis, Christopher S., Lyndsay M. Murrow, and Zev J. Gartner (Apr. 2019) "DoubletFinder: Doublet Detection in Single-Cell RNA Sequencing Data Using Artificial Nearest Neighbors". In: *Cell systems* 8 (4) 329–337.e4.

Riemondy, Kent A. et al. (July 2020) "clustifyr: an R package for automated single-cell RNA sequencing cluster classification". In: *F1000Research 2020 9:223* 9, p. 223.

Tirosh, Itay et al. (Apr. 2016) "Dissecting the multicellular ecosystem of metastatic melanoma by single-cell RNA-seq". In: *Science* 352 (6282) pp. 189–196.

Young, Matthew D. and Sam Behjati (Nov. 2020) "SoupX removes ambient RNA contamination from droplet-based single-cell RNA sequencing data". In: *GigaScience* 9 (12) pp. 1–10.

Zheng, Grace X.Y. et al. (Jan. 2017) "Massively parallel digital transcriptional profiling of single cells". In: *Nature Communications* 8 (1) pp. 1–12.

Supplementary Figures

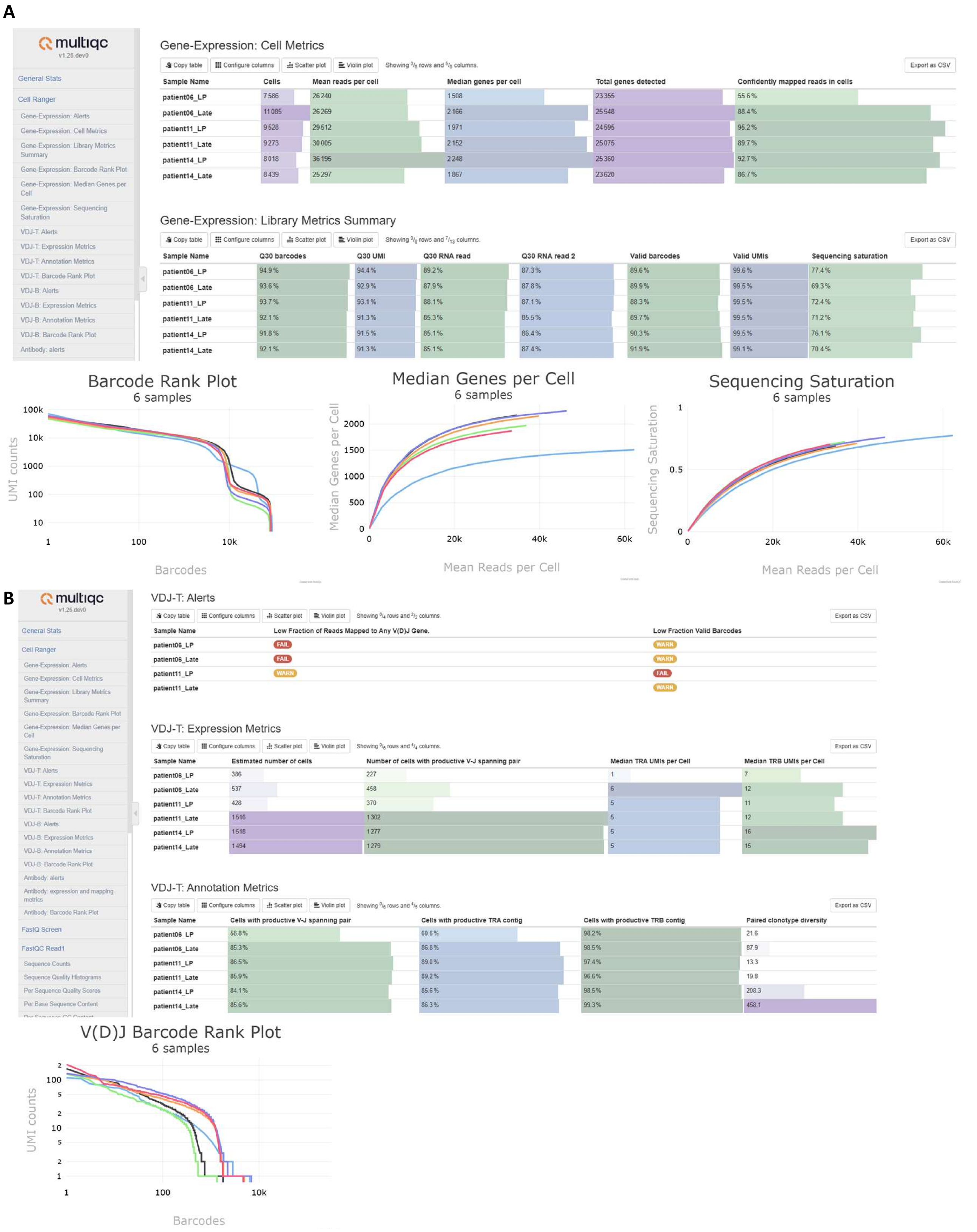

Fig. S1. Custom MultiQC module for 10x Genomics Cell Ranger outputs. The module aggregates key QC and performance metrics across samples for rapid, side-by-side comparison. **(A) Gene Expression (GEX):** cell metrics and library metrics, as well as barcode rank plot, median genes per cell and sequence saturation are given. **(B) V(D)J–T:** expression and mapping metrics, as well as V(D)J barcode rank plot is given. Alerts are shown when present.

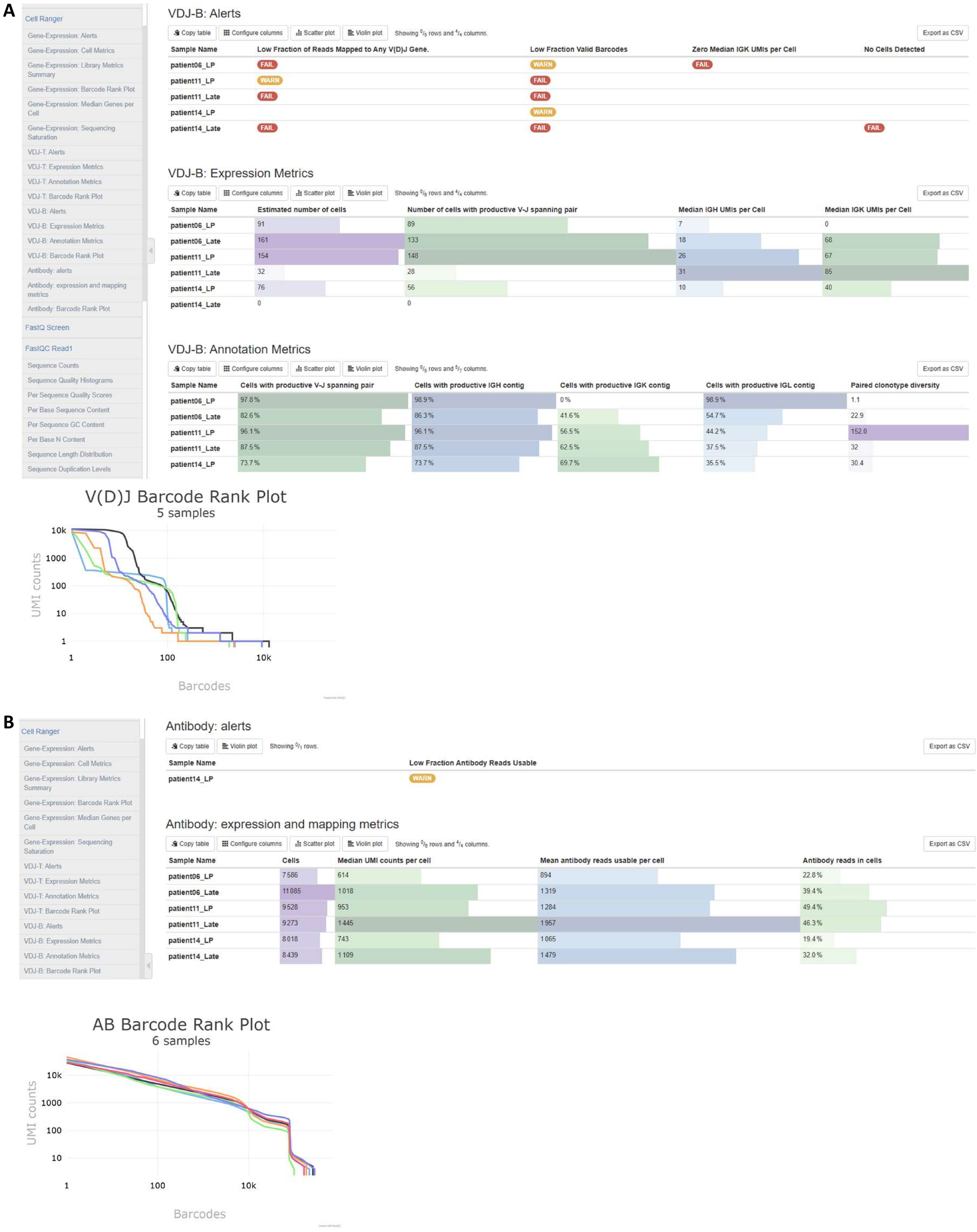

Sample Name	Low Fraction of Reads Mapped to Any V(D)J Gene.	Low Fraction Valid Barcodes	Zero Median IGK UMIs per Cell	No Cells Detected
patient06_LP	FAIL	WARN	FAIL	
patient11_LP	WARN	FAIL		
patient11_Late	FAIL	FAIL		
patient14_LP		WARN		
patient14_Late	FAIL	FAIL		FAIL

Sample Name	Estimated number of cells	Number of cells with productive V-J spanning pair	Median IGH UMIs per Cell	Median IGK UMIs per Cell
patient06_LP	91	89	7	0
patient06_Late	161	133	18	68
patient11_LP	154	148	26	67
patient11_Late	32	28	31	85
patient14_LP	76	56	10	40
patient14_Late	0	0		

Sample Name	Cells with productive V-J spanning pair	Cells with productive IGH contig	Cells with productive IGK contig	Cells with productive IGL contig	Paired clonotype diversity
patient06_LP	97.8%	98.9%	0%	98.9%	1.1
patient06_Late	82.6%	86.3%	41.6%	54.7%	22.9
patient11_LP	96.1%	96.1%	56.5%	44.2%	152.0
patient11_Late	87.5%	87.5%	62.5%	37.5%	32
patient14_LP	73.7%	73.7%	69.7%	35.5%	30.4

Sample Name	Low Fraction Antibody Reads Usable
patient14_LP	WARN

Sample Name	Cells	Median UMI counts per cell	Mean antibody reads usable per cell	Antibody reads in cells
patient06_LP	7586	614	894	22.8%
patient06_Late	11085	1018	1319	39.4%
patient11_LP	9528	953	1284	49.4%
patient11_Late	9273	1445	1957	46.3%
patient14_LP	8018	743	1065	19.4%
patient14_Late	8439	1109	1479	32.0%

Fig. S2. Custom MultiQC module for 10x Genomics Cell Ranger outputs (cont.). (A) V(D)J–B: expression and mapping metrics, as well as V(D)J barcode rank plot is given. **(B) Antibody (ADT):** expression and mapping metrics, as well as barcode rank plot is given. Alerts are shown when present.

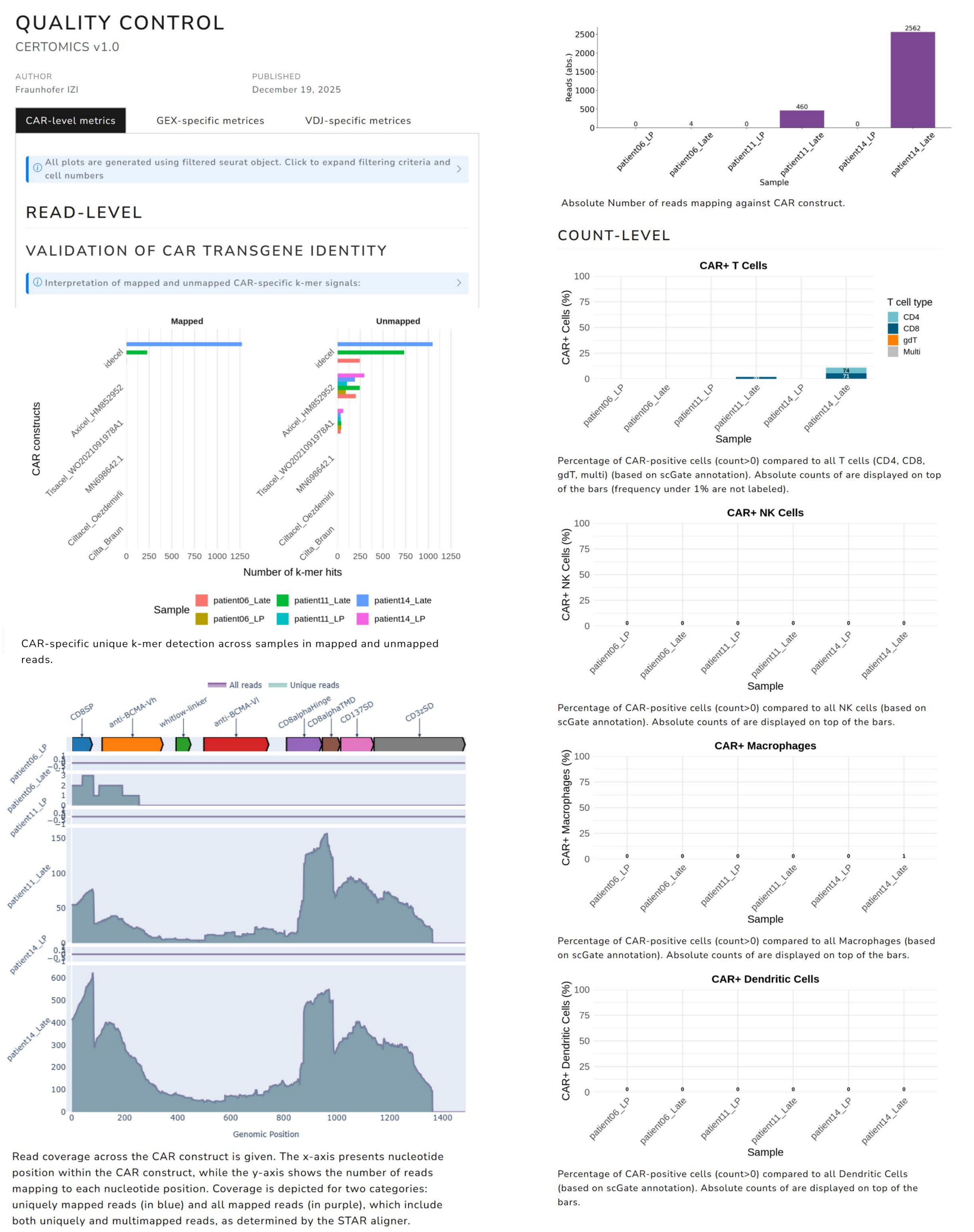

Fig. S3. Overview of interactive cross-sample quality control metrics displayed in an interactive webpage, enabling direct comparisons between different samples across three main categories (here: CAR, for GEX and V(D)J see S4): (left) Read level CAR-metrics including optional CAR identitiy step, read coverage of CAR construct (left) and absolute read counts (right). Count-level CAR-metrics across (CD4/CD8) T cells and other immune celltypes. The plot was cropped at the bottom due to space limitations.

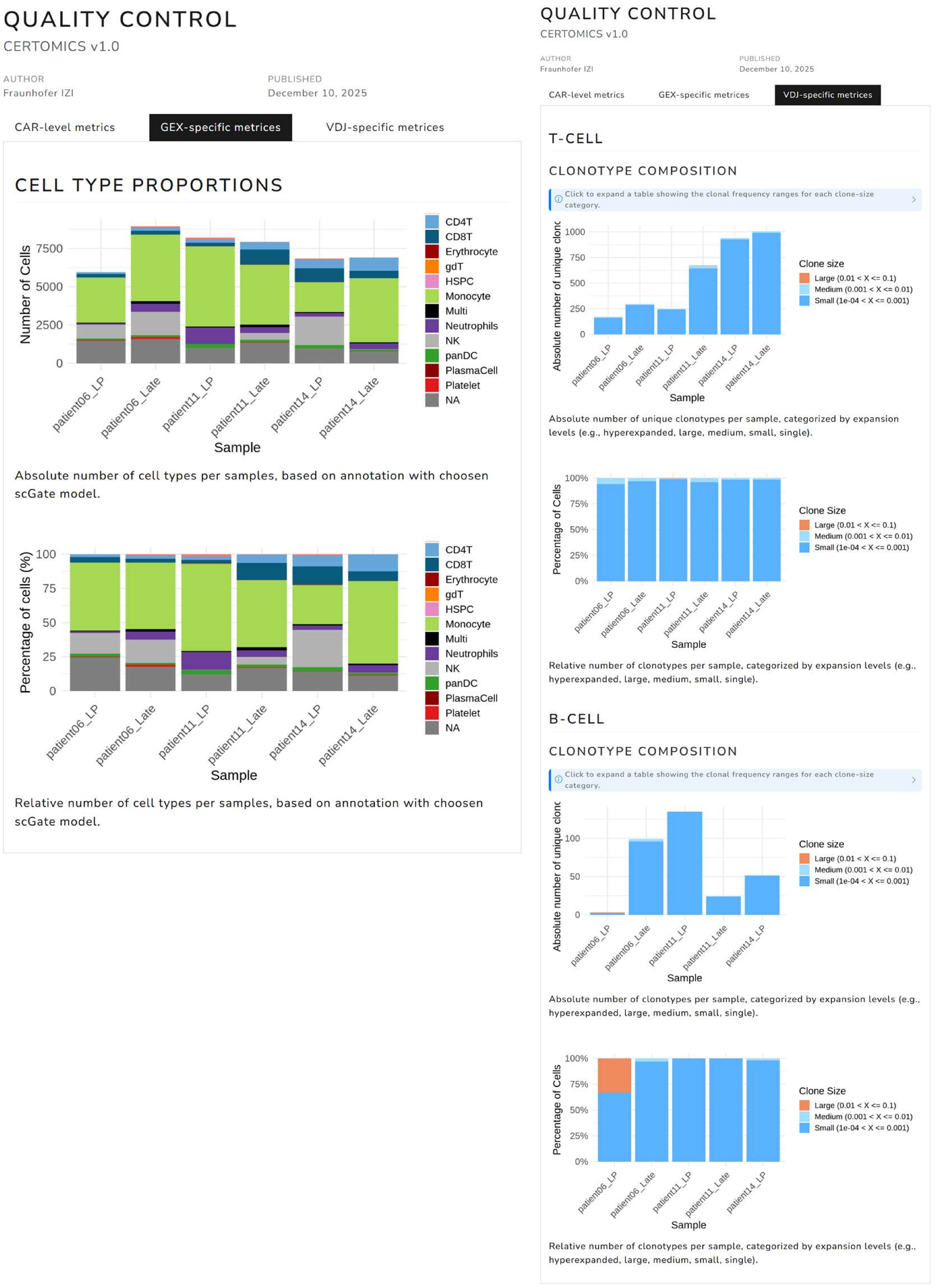

Fig. S4. Overview of interactive cross-sample quality control metrics displayed in an interactive webpage, enabling direct comparisons between different samples across three main categories (here: GEX and V(D)J, for CAR-metrics see S3): (left) GEX-specific metrics including absolute and relative proportions of annotated cell types per sample. (right) Absolute and relative clonotype composition of T cells and B cells. The plot was cropped at the bottom due to space limitations.

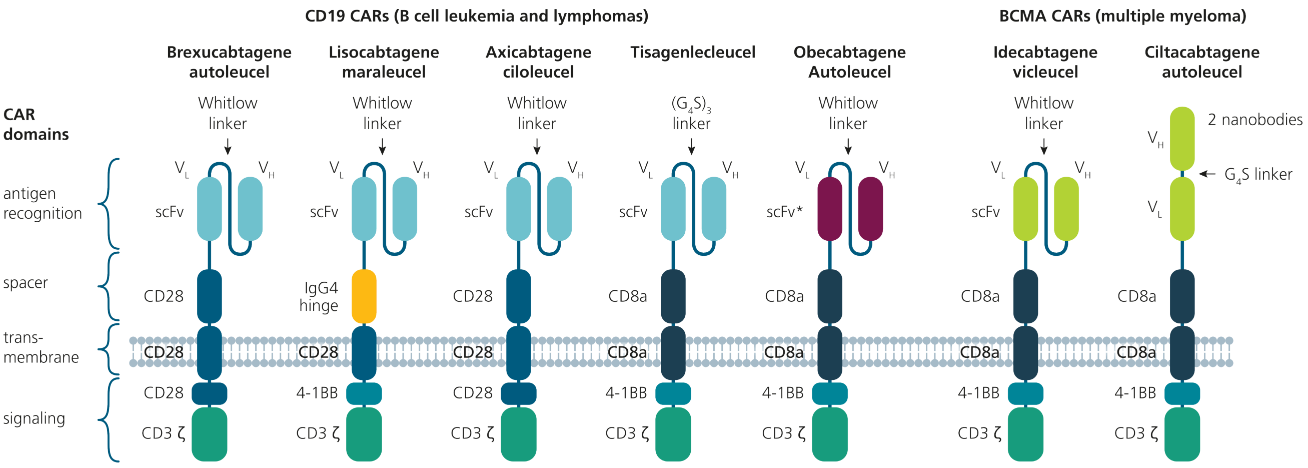

Fig. S5. Structural components of currently FDA-approved chimeric antigen receptor CAR T cell therapies targeting CD19 (B cell leukemia and lymphomas) and BCMA (multiple myeloma).

Supplementary Tables

Table S1. Sources of nucleotide and protein sequences for CAR constructs and associated vector systems in current CAR T cell therapies.

CAR T Cell Product	Original Source	CAR Sequence (DNA)	CAR Sequence (Protein)	Vector Sequence
**Ciltacel**	Patent *WO2022116086A1*	SEQ ID NO. 9–16	SEQ ID NO:17; translated from DNA	*Not available*
**Ciltacel**	Patent *US20230270786A1*	SEQ ID NO. 9–16	Seq ID NO 17; translated from DNA	*Not available*
**Ciltacel (Oezdemirli et al.)**	Supplementary Fig. S1 in Ozdemirli et al.	DNA sequence highlighted in blue	Translated from DNA	From 5'UTR to 3'UTR
**Ciltacel (Braun et al.)**	Braun et al. (2024)	Reverse engineered DNA provided at GitHub	Translated from reverse engineered DNA	Full reverse engineered vector sequence (same GitHub link)
**Idecel**	Patent *WO2021091978A1*	Sequence No. 10	Sequence No. 9; translated from DNA	Sequence No. 36
**Tisacel**	Patent *US 9,499,629 B2*	SEQ ID NO: 8	SEQ ID NO: 12; translated from DNA	SEQ ID NO: 1
**Axicel**	DrugBank via Roberts et al. → Kochenderfer et al. → GenBank ID *HM852952*	GenBank ID *HM852952*	Translated from DNA	*Not available*
